# Atypical cystic hepatorenal disease in a 40-year-old female: What is the diagnosis? A nephrology zebra

**DOI:** 10.1007/s40620-023-01728-3

**Published:** 2023-10-06

**Authors:** Frederic Barbey, Marie Maillard, Viviane Cina, Fadi Fakhouri

**Affiliations:** 1 Department of Medicine, Division of Immunology, Lausanne University Hospital and University of Lausanne, Av. du Bugnon 46, 1011 Lausanne, Switzerland; 2 Institute of Pathology, Lausanne University Hospital and University of Lausanne, Lausanne, Switzerland; 3 Division of Genetic Medicine, Lausanne University Hospital and University of Lausanne, Lausanne, Switzerland; 4 Service of Nephrology and Hypertension, Lausanne University Hospital and University of Lausanne, Lausanne, Switzerland

**Keywords:** Chronic kidney disease, Ciliopathies, Fibrocystic hepatorenal disease, Fibrocystin, Caroli disease

## Case description

**Fig. 1:**
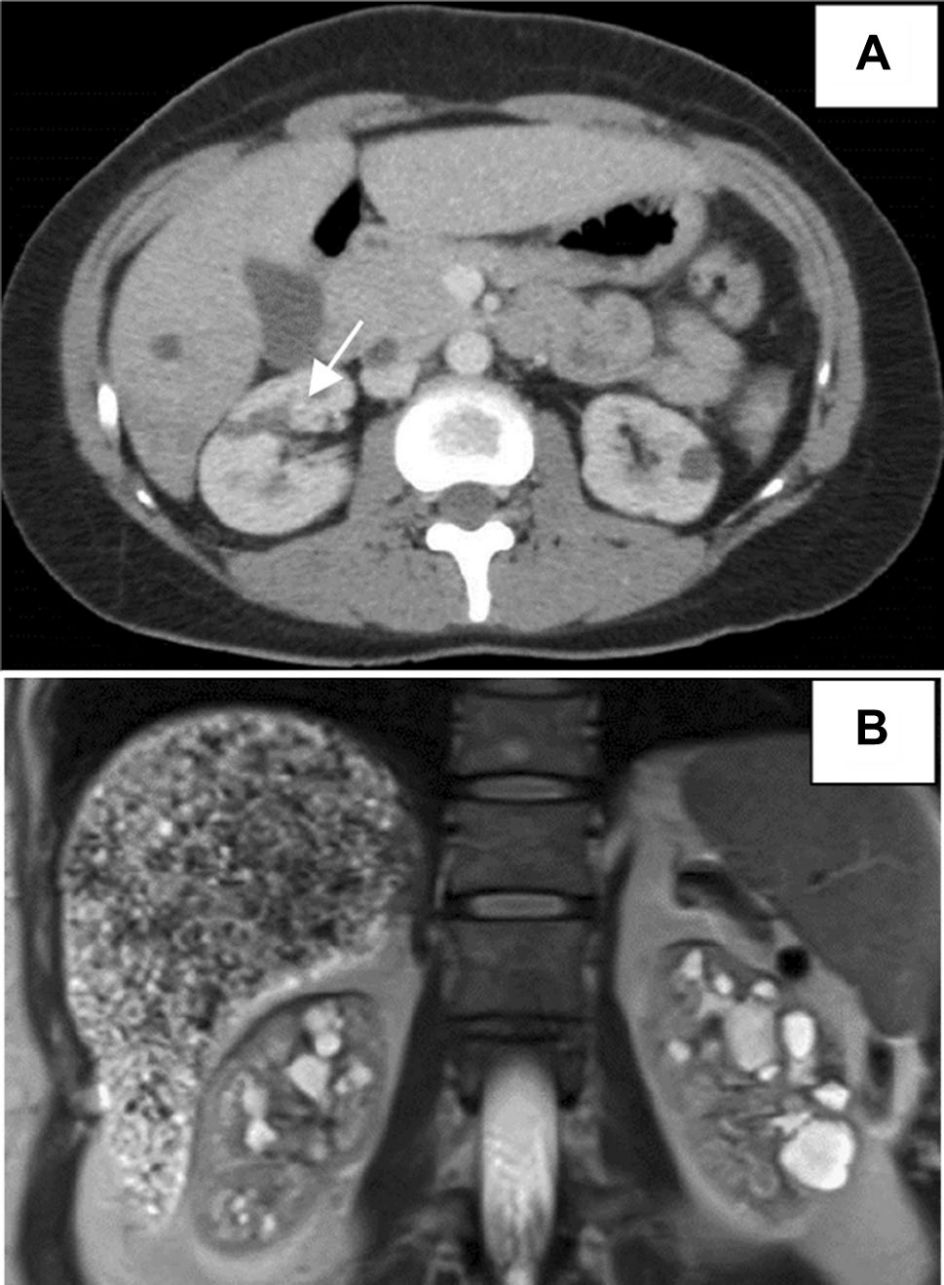
**A** Upper: axial computed tomography showing hypointense cysts of variable size in both kidneys and throughout the hepatic parenchyma. Microlithiasis (arrow) **B** Lower: T2-weighted magnetic resonance imaging: normal-sized kidneys with bilateral cysts located mainly at the cortico-medullary junction level and enlarged and heterogeneous liver containing multiple millimetric hyperintense lesions

In 2012, a 40-year-old woman was referred to our hospital following the incidental identification of renal and hepatic cysts via an abdominal computed tomography. This exam was performed due to recurring right flank pain. Her kidneys, although normal in size, contained multiple cysts of varying diameters and displayed signs of medullary microlithiasis (Fig. 1A). Aside from a few centimeter-sized cysts, her liver appeared unremarkable. Both the pancreas and internal genitalia were devoid of any anomalies.

Her past medical history included two episodes of renal colic which resolved spontaneously within 24 h, and one episode of acute *E. coli* pyelonephritis. Her blood pressure was normal. Laboratory testing revealed a serum creatinine level of 91 µmol/l, along with an estimated glomerular filtration rate (eGFR) of 71 ml/min per 1.73 m^2^, as calculated by the CKD-EPI (Chronic Kidney Disease Epidemiology Collaboration formula). Her blood counts, electrolytes, magnesium, glucose, uric acid, and liver test results were all within the normal range. Morning urinalysis demonstrated a pH of 6.5, hypocitraturia (0.71 mmol/L; normal ≥ 1 mmol/L), absence of crystalluria, and a protein-to-creatinine ratio of 35 mg/mmol. She had a family history of kidney stones affecting her father and two sisters, but there was no record of dialysis, renal transplantation, or liver disease. Renal ultrasound carried out on her parents yielded no significant findings. Given this atypical presentation, we debated the likelihood of a de novo autosomal dominant polycystic kidney disease (ADPKD) or an autosomal dominant tubulointerstitial kidney disease (*UMOD*, *MUC1*), as well as bilateral medullary sponge kidneys. However, we decided not to perform a kidney biopsy.

Over the following decade, her chronic kidney disease slowly progressed (with a mean decrease of eGFR: 2.2 ml/min per 1.73 m^2^ per year), without notable changes in kidney size or in the number of renal cysts (two follow-up abdominal ultrasounds at 41 and 43 years of age), proteinuria, or blood pressure. Similarly, liver size remained normal, and the number of cysts stayed consistent with a slightly heterogeneous parenchyma. By age 50, her creatinine level had increased to 115 µmol/l, and her eGFR dropped to 49 ml/min per 1.73 m^2^ (CKD-EPI). Liver function tests remained normal, except for a gamma-glutamyl transferase level of 85 U/I (normal range 6–42). Due to persisting, nonspecific right flank pain and the unexplained origin of chronic renal failure, abdominal magnetic resonance imaging was performed. This exam indicated no change in kidney size, but revealed numerous bilateral cysts predominantly situated at the cortico-medullary junction (Fig. 1B). The liver appeared enlarged and heterogeneous, containing cysts of various size. T2-weighted images also depicted multiple millimetric hyperintense lesions spread throughout the organ, and collateral circulation around the portal vein.

What is the diagnosis?

## Case solution

**Fig. 2:**
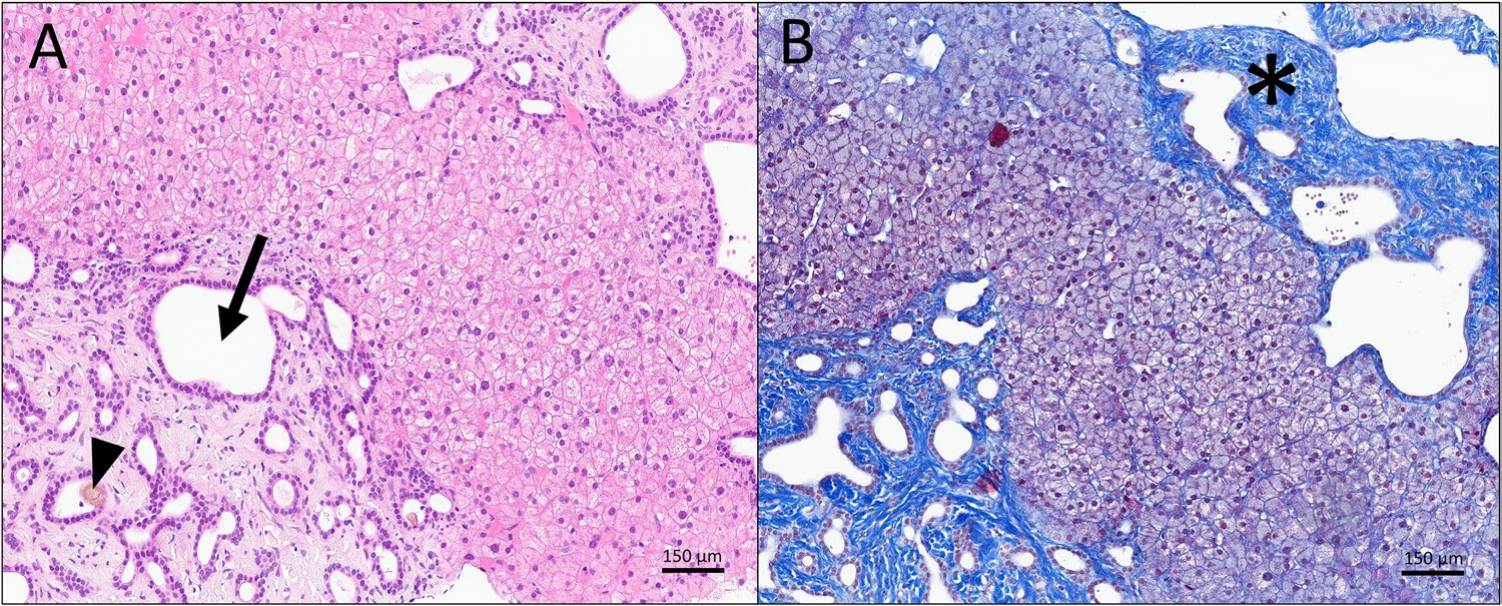
The Hematoxylin and Eosin (HE) stained liver biopsy showed numerous bile duct structures with irregular contours, often dilated (arrow), sometimes containing thickened bile in the lumen (arrowhead). Inflammation was minimal (**A**, HE, 100x). The trichrome stain highlighted the expanded portal tracts with extensive fibrosis (asterixis) (**B**, 100x)

Liver stiffness, assessed using transient elastography (FibroScan®), returned a high measurement of 47 kPa, considerably beyond the normal range of 2–7 kPa, suggesting the presence of cirrhosis. However, gastroscopy revealed no sign of portal hypertension. A comprehensive evaluation ruled out possibilities of viral hepatitis, autoimmune hepatitis, primary biliary cholangitis, alpha-1 antitrypsin deficiency, and Wilson's disease. A percutaneous liver biopsy ultimately disclosed a ductal plate malformation, characterized by portal tracts enlarged by dilated bile ducts and bridging fibrosis (Fig. 2). These manifestations are characteristic of congenital hepatic fibrosis, an abnormality typically associated with a diverse group of genetic disorders (ciliopathies), including autosomal recessive polycystic kidney disease (ARPKD) and Caroli syndrome. An analysis of the *PKHD1* gene, whose mutations are linked to ARPKD, revealed two heterozygous missense mutations c.3383 T > C (p.Ile1128Thr) and c.11210A > G (p.Tyr3737Cys). The first variant is recognized as pathogenic, and the second as likely pathogenic, thus confirming a late diagnosis of autosomal recessive polycystic kidney disease in our patient [1].

ARPKD is the most common childhood-onset hepatorenal cystic disorder, with an estimated incidence of 1 in 20,000 live births. The majority of cases are identified either in utero or at birth, due to enlarged echogenic kidneys, which often result in pulmonary hypoplasia and perinatal mortality [2]. Patients who survive the first year often develop kidney or liver-related symptoms in childhood, with half of them progressing to end-stage renal disease within the first decade of life. Despite being primarily a childhood disease, ARPKD can also be diagnosed in adults, presenting as an ADPKD-like phenotype with a slow eGFR decrease (less than 3 ml/min/year) without progressive kidney enlargement. Alternatively, the disease may manifest with a liver-predominant phenotype, including portal hypertension with mild or no evident kidney disease. However, adult diagnoses of ARPKD are exceedingly rare. The condition invariably associates a cystic renal disease and congenital liver fibrosis, which may or may not be accompanied by dilated bile ducts (Caroli syndrome) [3].

ARPKD is a cilia-related disease, typically caused by biallelic pathological variants in the *PKHD1* gene (or rarely, the *DZIP1L* gene). The latter gene codes for fibrocystin, a transmembrane protein of unknown cellular function present mostly in adult renal collecting ducts and bile ducts. Genotype–phenotype correlations indicate that the risk of perinatal demise is predominantly associated with two truncating or null variants. Children with at least one missense mutation (hypomorphic allele) generally experience less severe disease, a wider range of clinical variability, and longer life expectancy. Additionally, the affected region of the *PKHD1* gene contributes to the broad phenotypic spectrum of ARPKD [4]. Patients with one or two missense variants affecting amino acids 709–1837 typically exhibit a better renal prognosis, while those with variants affecting amino-acids 2625–4074 tend to have a poorer hepatic outcome [5].

Given the advances in CKD management, the long-term prognosis primarily depends on the severity of hepatobiliary complications (cholangitis, portal hypertension). For late-presenting cases, the diagnosis of ARPKD can be a challenge due to an atypical radiological pattern, as exemplified by our patient.

## Data Availability

The full report of our patient's paraclinical examinations are available from the corresponding author as reasonable request.

## References

[1] Capuano I, Buonanno P, Riccio E (2022). Parapelvic cysts: an imaging marker of kidney disease potentially leading to the diagnosis of treatable rare genetic disorders? A narrative review of the literature. J Nephrol.

[2] Gunay-Aygun M, Font-Montgomery E, Lukose L (2010). Correlation of kidney function, volume and imaging findings, and PKHD1 mutations in 73 patients with autosomal recessive polycystic kidney disease. Clin J Am Soc Nephrol.

[3] Gunay-Aygun M, Font-Montgomery E, Lukose L (2013). Characteristics of congenital hepatic fibrosis in a large cohort of patients with autosomal recessive polycystic kidney disease. Gastroenterology.

[4] Burgmaier K, Brinker L, Erger F (2021). Refining genotype-phenotype correlations in 304 patients with autosomal recessive polycystic kidney disease and PKHD1 gene variants. Kidney Int.

[5] Rizzo M, Pezone I, Amicone M (2023). Familial polycystic kidneys with no genetic confirmation: Are we sure it is ADPKD?. Clin Nephrol.

